# Crystal structure and epitope analysis of house dust mite allergen Der f 21

**DOI:** 10.1038/s41598-019-40879-x

**Published:** 2019-03-20

**Authors:** Sze Lei Pang, Kok Lian Ho, Jitka Waterman, Robert Paul Rambo, Aik-Hong Teh, Indran Mathavan, Gemma Harris, Konstantinos Beis, Yee-How Say, Matta Sri Anusha, Yang Yie Sio, Fook Tim Chew, Chyan Leong Ng

**Affiliations:** 1 Institute of Systems Biology, Universiti Kebangsaan Malaysia, 43600 UKM Bangi, Selangor, Malaysia; 2 Department of Pathology, Faculty of Medicine and Health Sciences, Universiti Putra Malaysia, 43400 UPM Serdang, Selangor, Malaysia; 3 Diamond Light Source, Harwell Science & Innovation Campus, Didcot, Oxfordshire OX11 0DE UK; 4 Centre for Chemical Biology, Universiti Sains Malaysia, 10 Persiaran Bukit Jambul, 11900 Bayan Lepas, Penang Malaysia; 5 Department of Life Sciences, Imperial College London, South Kensington, London SW7 2AZ United Kingdom; 6 Research Complex at Harwell, Rutherford Appleton Laboratory, Oxfordshire, OX11 0FA United Kingdom; 7 Department of Biomedical Science, Faculty of Science, Universiti Tunku Abdul Rahman (UTAR) Perak Campus, 31900 Kampar, Perak, Malaysia; 8 Department of Biological Sciences, National University of Singapore, 14 Science Drive 4, 117543 Singapore, Singapore

## Abstract

Group 21 and 5 allergens are homologous house dust mite proteins known as mid-tier allergens. To reveal the biological function of group 21 allergens and to understand better the allergenicity of the rDer f 21 allergen, we determined the 1.5 Å crystal structure of rDer f 21 allergen from *Dermatophagoides farinae*. The rDer f 21 protein consists of a three helical bundle, similar to available structures of group 21 and homologous group 5 allergens. The rDer f 21 dimer forms a hydrophobic binding pocket similar to the one in the Der p 5 allergen, which indicates that both of the homologous groups could share a similar function. By performing structure-guided mutagenesis, we mutated all 38 surface-exposed polar residues of the rDer f 21 allergen and carried out immuno-dot blot assays using 24 atopic sera. Six residues, K10, K26, K42, E43, K46, and K48, which are located in the region between the N-terminus and the loop 1 of rDer f 21 were identified as the major IgE epitopes of rDer f 21. Epitope mapping of all potential IgE epitopes on the surface of the rDer f 21 crystal structure revealed heterogeneity in the sIgE recognition of the allergen epitopes in atopic individuals. The higher the allergen-sIgE level of an individual, the higher the number of epitope residues that are found in the allergen. The results illustrate the clear correlation between the number of specific major epitope residues in an allergen and the sIgE level of the atopic population.

## Introduction

Nearly three billion people, about 40% of the world population, are affected by immunoglobulin E (IgE) antibody sensitization against foreign proteins present in the environment^1^. Exposure to allergen proteins from house dust mites (HDMs) is one of the major contributors to allergy sensitization^1^, affecting 45–85% of asthmatics in Europe, North and South America, Africa, Asia and Pacific^2^. The geographical distribution of HDMs varies with *Dermatophagoides farinae* (Der f) and *Dermatophagoides pteronyssinus* (Der p) being the most prevalent in temperate countries, whereas *Blomia tropicalis* (Blo t) is predominant in tropical and subtropical regions^3–6^. To date, a total of 36 groups of HDM protein allergens have been identified and accepted by World Health Organization/International Union of Immunological Societies Subcommittee of Allergen Nomenclature. Among these, group 21 allergens including Blo t 21^7^, Der p 21^8^ and Der f 21^9^ identified in *B*. *tropicalis*, *D*. *pteronyssinus* and *D*. *farinae*, respectively, are known as mid-tier allergens in the allergic response.

Structural biology of allergens forms the basis for the IgE epitope mapping and rational design of hypoallergens with reduced IgE binding^10^. The structural approach has also provided an insight into the biological function of allergens through bound ligands and thus into the understanding of the protein allergenicity^11–13^. Structural analysis has previously been used to identify the antigenic determinants and to further clarify the allergenicity of group 21 and the homologous group 5 of the HDM allergens. Comparison of the NMR structures of Blo t 5 from *B*. *tropicalis* (Protein Data Bank [PDB] codes 2JMH and 2JRK)^14,15^ and Blo t 21 (PDB code 2LM9)^16^ with the crystal structure of Der p 5 (PDB code 3MQ1)^12^ shows all available allergen structures from groups 21 and 5 share a common structural fold consisting of a helical bundle that is formed by three anti-parallel α-helices. Nonetheless, the biological function of group 21 and 5 allergens remains to be identified.

In current allergen diagnostics, molecular allergy diagnostics (MAD) using recombinant allergenic molecules in singleplex and multiplex assays has improved the sensitivity and specificity of the specific IgE antibodies determination^17^. MAD also allows for a more accurate diagnosis with consideration of the cross-reactivity of homologous allergens^18^. In MAD development of the HDM allergens, detection of major allergens including Der f 1 and 2, Der p 1 and 2, Der p 10, *Lepidoglyphus destructor* Lep d 2 and Blo t 5 has been established and is commercially available (ImmunoCAP^®^ ISAC 112, Thermofisher, Uppsala, Sweden). Studies focused on improving the detection sensitivity of minor allergens are under way. To improve diagnosis of allergy and allergen-specific immunotherapy treatment, most of the studies have been focused on identifying the major allergen epitopes. Recently, the correlation between IgE titre and IgE-repertoire complexity was demonstrated by accessing the complex formation between IgE, Der p 2 allergen, and CD23 on a B cell^19^. Sera of increased Der p 2-specific IgE (sIgE) titres were shown to correlate with increased complexity of the IgE repertoires^19^. Since there is a lack of comprehensive data concerning how each of the single solvent-accessible polar/charged residues located on the surface of an aeroallergen contributes to the sIgE-allergen interaction, we therefore mapped all the surface-exposed polar/charged residues of recombinant Der f 21 (rDer f 21) and conducted site-directed mutagenesis to generate 38 single-point mutants for immuno-dot blot assay using 24 atopic sera. Our aim was to understand the sIgE interaction profile towards an allergenic molecule at the single residue level and also to investigate whether there is a correlation between the sIgE level of an atopic population and the number of major epitope residues that sIgE can recognize for an aeroallergen.

Here, we report the crystal structure of rDer f 21 at 1.5 Å resolution. We mapped all surface-exposed polar and charged residues on the structure to guide the single residue mutagenesis of rDer f 21. Mutant proteins were produced and purified for IgE-binding epitope analysis. The major epitope residues of rDer f 21 were identified for this sera population. Comprehensive immuno-dot blot analysis revealed that the rDerf21-sIgE level of atopic individuals is positively correlated to the variants of sIgE that are found in the individuals and to the number of major epitope residues that are recognised by sIgE. These findings suggest that individuals with high allergen-sIgE levels may face a bigger challenge in personal immunotherapy. Comprehensive allergen-sIgE screening tests that include all surface-exposed residues of an allergen may help to improve the accuracy, specificity and sensitivity of future allergen diagnosis and personal allergen-specific immunotherapy treatments.

## Results

### Overall structure of rDer f 21

**Figure 1:**
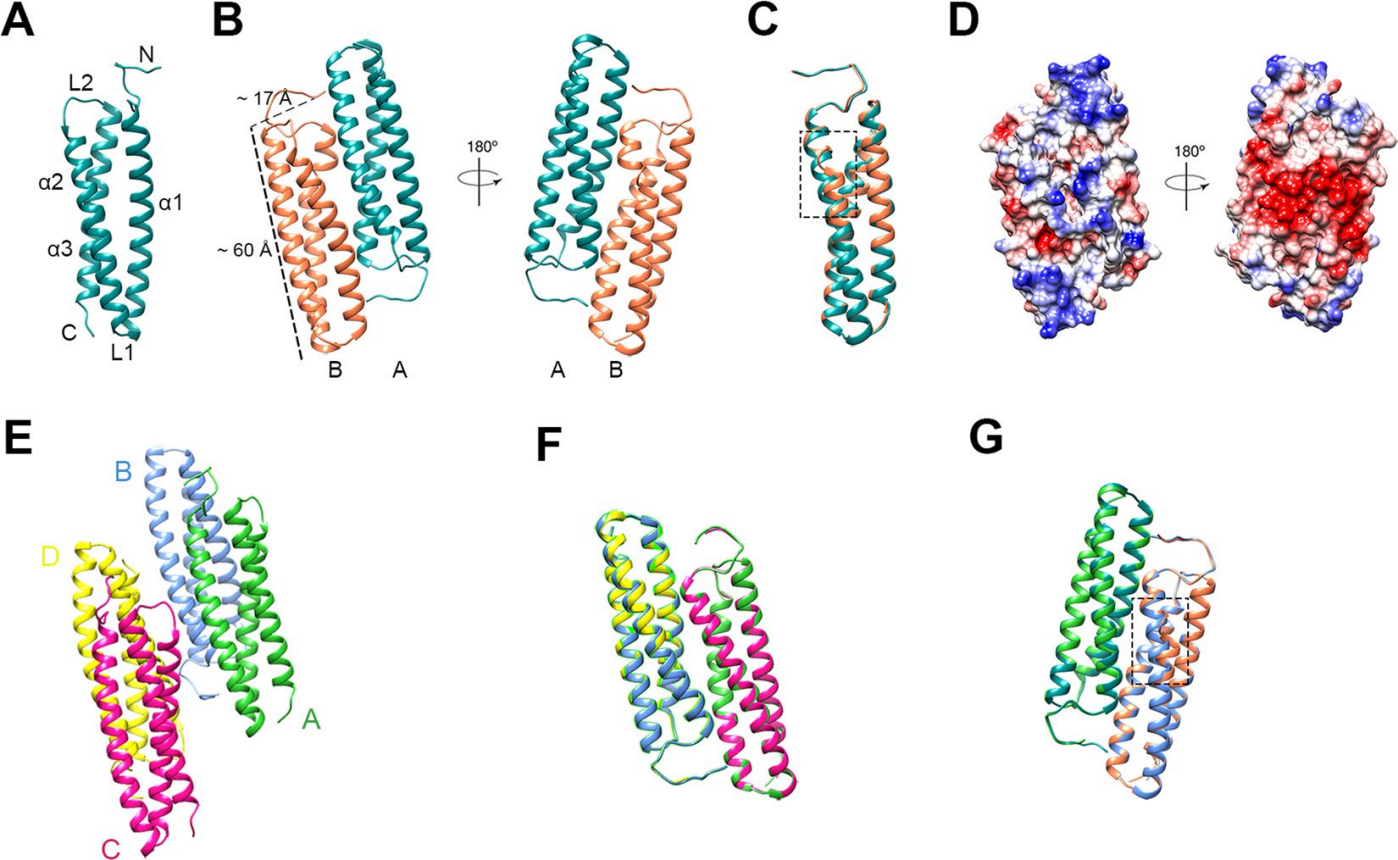
Crystal structure of rDer f 21. (**A**) Molecule A of the dimer presents in the rDer f 21^PEG400^ structure. The N-terminus, the C-terminus, the α-helices (α1, α2 and α3) and the loops (L1 and L2) are marked. (**B**) Two orientations of the rDer f 21^PEG400^ dimer. There are two molecules per asymmetric unit of the crystal grown in precipitant PEG 400 (molecule A is in cyan and molecule B is in orange). (**C**) Superimposition of molecules A and B of the rDer f 21^PEG400^ dimer shows r.m.s.d. of 0.9 Å. A significant structural deviation is localised at the helix α1 of the molecule B between residues 20-30 (dashed line box). (**D**) Two orientations of the rDer f 21^PEG400^ electrostatic surface diagram as calculated by the program Chimera^53^. (**E**) Structure of rDer f 21^PEG2KMME^ dimers. There are four molecules forming two dimers in the asymmetric unit of the crystal grown in precipitant PEG 2000 MME. (**F**) Superimposition of AB and CD dimers of the rDer f 21^PEG2KMME^. (**G**) Superimposition of molecules A and B of both of the AB dimers from the rDer f 21^PEG400^ and rDer f 21^PEG2KMME^ structures. Significant structural deviation of molecule B is boxed with a dashed line. The figures were generated using the program Chimera^54^.

The allergen protein rDer f 21, consisting of 128 amino acid residues, was cloned and purified with a 6x-His-tag at the N-terminus^20^. The structures of rDer f 21 were obtained from two different crystal forms grown using either PEG 400 or PEG 2000 MME as a precipitant. The structure of rDer f 21 bound to a molecule of PEG 400, rDer f 21^PEG400^, was determined to 1.5 Å resolution (Fig. 1A–D) from a crystal belonging to C-centered monoclinic space group C2. The structure contains two molecules per asymmetric unit forming a rDer f 21 dimer (Table 1). The similar dimeric form of rDer f 21 protein crystal structure grown from the solution containing PEG 2000 MME, rDer f 21^PEG2KMME^, was solved to 2.3 Å resolution (Fig. 1E–G). This crystal contains four molecules per asymmetric unit, which represent two dimers of rDer f 21. The structure of rDer f 21 adopts a bundle of three anti-parallel α-helices similar to the group 21 HDM allergen Blo t 21^16^, and group 5 HDM allergens including Blo t 5^14,15^ and Der p 5^12^. The rDer f 21^PEG400^ and rDer f 21^PEG2KMME^ structures show high similarity with a root mean square deviation (r.m.s.d.) of 0.7 Å over 231 Cα atoms.

**Table 1:** Crystallographic data.

	rDer f 21^PEG400^	rDer f 21^PEG2KMME^
Wavelength (Å)	1.5418	0.9795
Resolution range (Å)	19.60–1.49 (1.52–1.49)	28.74–2.30 (2.38–2.3)
Space group	C2	P2_1_
**Unit cell**		
a, b, c (Å)	123.5, 27.7, 90.3	63.2 50.9 69.6
α, β, γ (°)	90.0, 125.8, 90.0	90, 97.3, 90.00
Measured reflections	135705	71129
Unique reflections	41016	19287
R_sym_	0.031 (0.320)	0.045 (0.476)
CC (1/2)	1.0 (0.977)	0.999 (0.903)
Mean I/σI (I)	22.6 (3.1)	16.2 (3.3)
Multiplicity	3.3 (2.5)	3.7 (3.7)
Mosaicity (°)	0.14	0.16
Completeness (%)	99.5 (95.6)	97.8 (98.1)
**Refinement statistics**		
R_cryst_, R_free_ (%)	13.9, 19.8	22.1, 27.3
No. of molecules per asymmetric unit	2	4
No. of water molecules	261	58
No. of glycerol molecules	2	—
No. of polyethylene glycol (PEG) 400 molecules	1	—
No. of β-mercaptoethanol molecules	1	—
**Root mean square deviation from ideal values (r**.**m**.**s**.**d**.**)**		
Bond length (Å)	0.019	0.014
Bond angle (°)	1.85	1.59
**Ramachandran plot statistics**		
Favoured regions (%)	100	99.09
Allowed regions (%)	0	0.91
Average B factors (All atoms)	32	71

R_sym_ = Ʃ(ǀ*I*_*i*_ − <I>ǀ)/Ʃ(*I*_*i*_), where *I*_*i*_ is the intensity of the *i*th measurement and <I> is the mean intensity of that reflection.

R_cryst_ = Ʃǀǀ*F*_o_ǀ − ǀ*F*_c_ǀǀ/Ʃǀ*F*_o_ǀ calculated from the working data set.

R_free_ was calculated from 5% of data randomly chosen not to be included in refinement.

Each of the two subunits of rDer f 21^PEG400^ consists of 117 residues, which includes seven amino acid residues (G-L-V-P-R-G-S) from the fusion tag of the pET M vector at the N-terminus, followed by 110 residues (E8 to S117) of the Der f 21 protein (according to the numbering of GenBank Accession Number AHC 94806)^9^. No electron density was observed for the first nine N-terminal residues from pET-M and the last two residues of the C-terminus, presumably due to the flexibility of these regions. Each molecule of rDer f 21^PEG400^ is approximately 60 × 17 × 14 Å and forms a coiled coil bundle structure composed of three anti-parallel α-helices denoted as α1 (A14 to T47), α2 (K50 to A78), and α3 (M85 to K113). The α-helices are connected by loops L1 (K48 to S49) and L2 (K79 to N84) (Fig. 1A). Helices α1 and α2 form an intermolecular anti-parallel dimeric interface while the C-terminal α-helix (α3) is fully exposed to the solvent.

The crystal structure of rDer f 21 revealed an elongated dimer structure with molecules A and B interacting in a head-to-toe orientation (Fig. 1B). The dimer forming molecules A and B have an r.m.s.d of 0.9 Å over 117 Cα atoms (Fig. 1C). The Proteins Interfaces Structures and Assemblies (PDBePISA) program^21^ calculated an interface score of 0.7 with an interacting interface area of 1245 Å^2^ (~16% of the total surface area), suggesting that the dimer is probably a biological assembly. At the dimer interface, 11 inter-helical hydrogen bonds and two salt bridges are formed between helices α1 and α2 of molecules A and B (Supplementary Table S1). Electrostatic surface analysis using the program Chimera revealed that the rDer f 21 protein surface consists of mostly negatively-charged residues in one orientation, while the other orientation (180° rotation) has an even distribution of polar residues (Fig. 1D). Previous studies have shown that the HDM group 5 and 21 allergen proteins also have a high distribution of charged residues on their surface^12,15,16^. Further interface analysis revealed that the side-chain of residue R5^A^ (arginine 5 of molecule A), which belongs to the 6x-His-tag fusion of rDer f 21^PEG400^, forms a hydrogen bond with the side-chain of residue Q55^B^ (residue glutamine 55 of molecule B) at distance of ~3.1 Å. Q55^B^ also forms a hydrogen bond with the side-chain of Asn13^A^, resulting in a triple hydrogen bond network among R5^A^, N13^A^ and Q55^B^. The side-chain of arginine R5^A^ forms a stacking interaction with the side-chains of the residues R12^A^ and R58^B^. Similar interactions were also observed for the side-chain of residue R5 of molecule B. These interface interactions originating from residue R5 might have artificially induced the dimer formation of rDer f 21 (Supplementary Fig. S1). To better understand the conformation of Der f 21, therefore, we produced rDer f 21 with the His-tag cleaved, (crDer f 21) for SEC, chemical cross-linking, SLS, SEC-MALS and SAXS analyses and showed that Der f 21 exists as both a monomer and a dimer in a concentration dependent manner (see section below).

**Figure 2:**
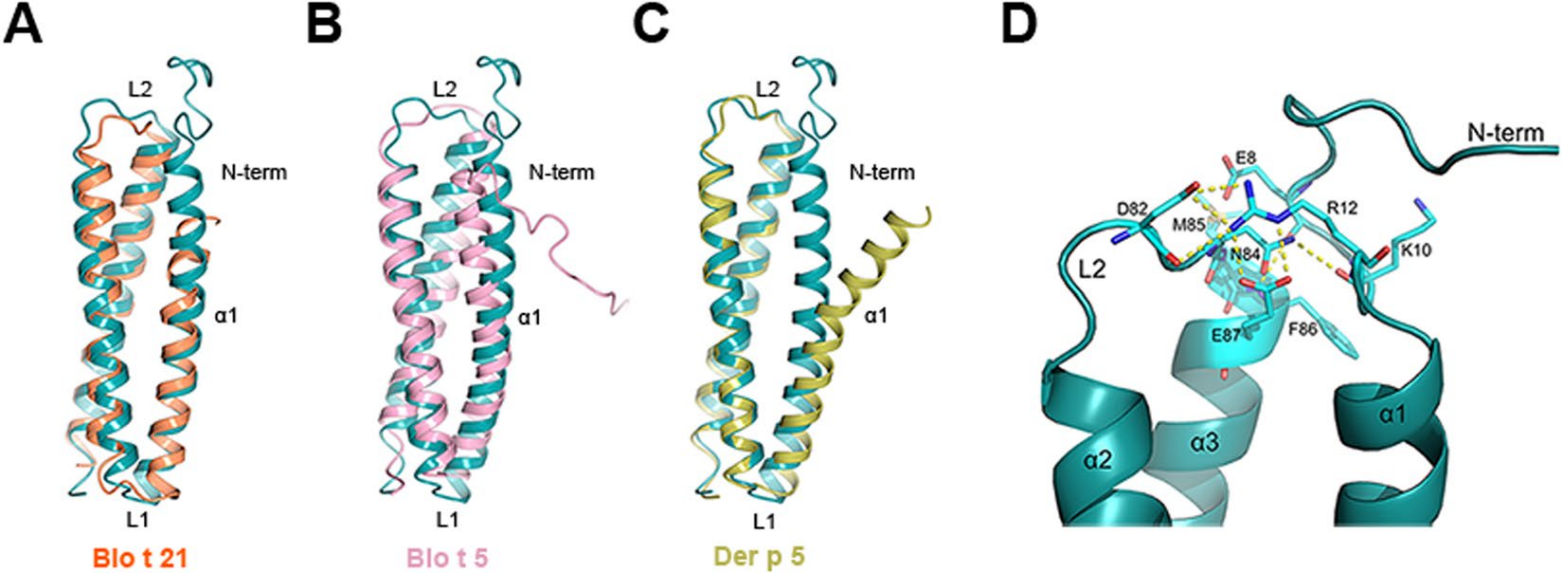
Superimposition of the rDer f 21 structure (cyan) and (**A**) Blo t 21 (PDB code 2LM9) (orange) shows r.m.s.d. 2.5 Å over 98 Cα atoms, (**B**) Blo t 5 (PDB code 2JMH) (pink) shows r.m.s.d. 2.5 Å over 95 Cα atoms, and (**C**) Der p 5 (PDB code 3MQ1) (gold) shows r.m.s.d. 1.0 Å over 77 Cα atoms. (**D)** The network of the hydrogen bond interactions formed between the N-terminus, L2 loop region and helix α3 of rDer f 21. The figures were generated using the molecular-graphics program CCP4mg^55^.

The overall structure of rDer f 21 is similar to its homologues from group 21 and 5 allergens including Blo t 21^16^, Blo t 5^14^ and Der p 5^12^. While the helices α2 and α3 from rDer f 21, Blo t 21, Blo t 5, and Der p 5 show a substantial similarity, a significant structural discrepancy was observed at helix α1, especially in the N-terminal region and both loops L1 and L2 (Fig. 2A–C). The N-terminus of rDer f 21 (with fusion tag residues excluded) that forms helix α1 was found to have the longest α1 helix compared to all available homologous structures (Fig. 2A–C). In addition, the unstructured N-terminal region (residues 8–13) adjacent to the α1 helix interacts with the helix α3 via a hydrogen bond between main-chain O atom of E8 and the main-chain N atom of F86 (3.0 Å), and via a hydrogen bond between the side-chain of E8 and the main-chain N atom of M85 (2.9 Å), whereas both the main-chain and side-chain of R12 interact via hydrogen bonds with the side-chain of E87 (Fig. 2D). Furthermore, additional hydrogen bonds are formed between K10-N84 and R12-D82 in the L2 loop region. These observations show for the first time that the N-terminal region of group 5 and 21 allergens can interact tightly with helix α3 and the L2 loop region.

**Figure 3:**
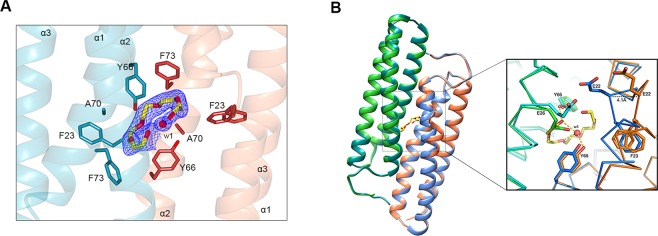
The rDer f 21 structure undergoes conformational changes with the distortion of the helix α1 upon binding of PEG 400 in the hydrophobic cavity formed in the rDer f 21 dimer interface. (**A**) The PEG 400 molecule is bound between helices α1 and α2 of molecules A (cyan) and B (coral), and is surrounded with hydrophobic residues, i.e. F23 (α1), A70 (α2), Y66 (α2), and F73 (α2). A water molecule (w1) mediates the interaction between the PEG400 ligand and Tyr66 from the structure. The 2Fo - Fc electron density map of the PEG 400 and water molecule bound at the dimer interface of Der f 21^PEG400^ contoured at 1.0 *σ*. (**B**) Superimposition of the dimers AB from the rDer f 21^PEG400^ (molecule A in cyan and B in orange) and rDer f 21^PEG2KMME^ (molecule A in green and B in blue) structures shows the biggest conformational changes at the region of the residues 21–26 region (box). Compared to the undistorted rDer f 21^PEG2KMME^ structure, residues 20–30 of the α1 helix of molecule B of rDer f 21^PEG400^, especially residue E22, undergoes a dramatic conformation change in order to accommodate the PEG 400 ligand in the cavity. The figures were generated using the molecular-graphics program CCP4mg^55^ and Pymol^56^.

Structural comparison of two molecules A and B representing the asymmetric unit of the rDer f 21^PEG400^ crystal structure shows a significant structural deviation localised at the α1 helix of molecule B between residues 20-30. The r.m.s.d. between the two molecules is reaching up to 4.3 Å (Fig. 1C). The deviation was also observed when the dimer AB of rDer f 21^PEG400^ was compared with both the dimers AB (Fig. 1G) and CD of rDer f 21^PEG2KMME^. The dramatic conformational changes at the helix α1 of the molecule B are caused by the binding of a well-ordered PEG 400 molecule (from the crystallization buffer) in the pocket of the rDer f 21^PEG400^ molecule (Fig. 3A). The main-chain C atoms of E22 moved by ~4.1 Å and a significant conformational change is also observed for the side-chain of the residue E22 (molecule A) (Fig. 3B). Interestingly, the coordinates of other residues surrounding the ligand binding pocket including F23 and Y66 are well conserved. The conformational changes enable binding of the ligand into the dimer interface cavity, an event which may otherwise be prevented by steric clashes (Fig. 3B).

The binding cavity consists mostly of hydrophobic residues including A70 and three aromatic residues F23, F73, and Y66 (Fig. 3A). This indicates that the binding pocket can accommodate a hydrophobic ligand. The Y/F aromatic residues 66 and 73 are conserved across all group 21 HDMs allergens (Supplementary Fig. S2), suggesting that a similar ligand binding pocket may also exist in other dimeric proteins from the group 21 allergens. A similar lipid binding site has also been proposed previously for the group 5 allergen Der p 5^12^. The PEG 400 molecule in the cavity of the rDer f 21 dimer interface only forms one hydrogen bond to Y66^A^ (residue Y66 of molecule A) and is further coordinated via several hydrogen bonds with water molecules in the cavity (Fig. 3B). A water molecule (w1) coordinates at the middle of the PEG 400 molecule, which adopted an incomplete circular shape. w1 also mediates the interaction between Y66^B^ and PEG 400 (Fig. 3B). The lack of direct hydrogen bonds between the PEG 400 molecule and the surrounding residues suggests that the cavity would prioritize a hydrophobic ligand. In addition, unidentified regions of small molecule electron density were also found in the dimer interface cavities formed by hydrophobic residues A14, F15, M18 (Molecule A/B) and G33, M33, L37 (Molecule B/A) of the rDer f 21^PEG400^ (Supplementary Fig. S3). Although this observation prompted us to hypothesize that Der f 21 may contain a functional ligand cavity, the Der f 21 dimer was only formed at high protein concentrations as described below.

### Der f 21 exists as both a monomer and a dimer

**Figure 4:**
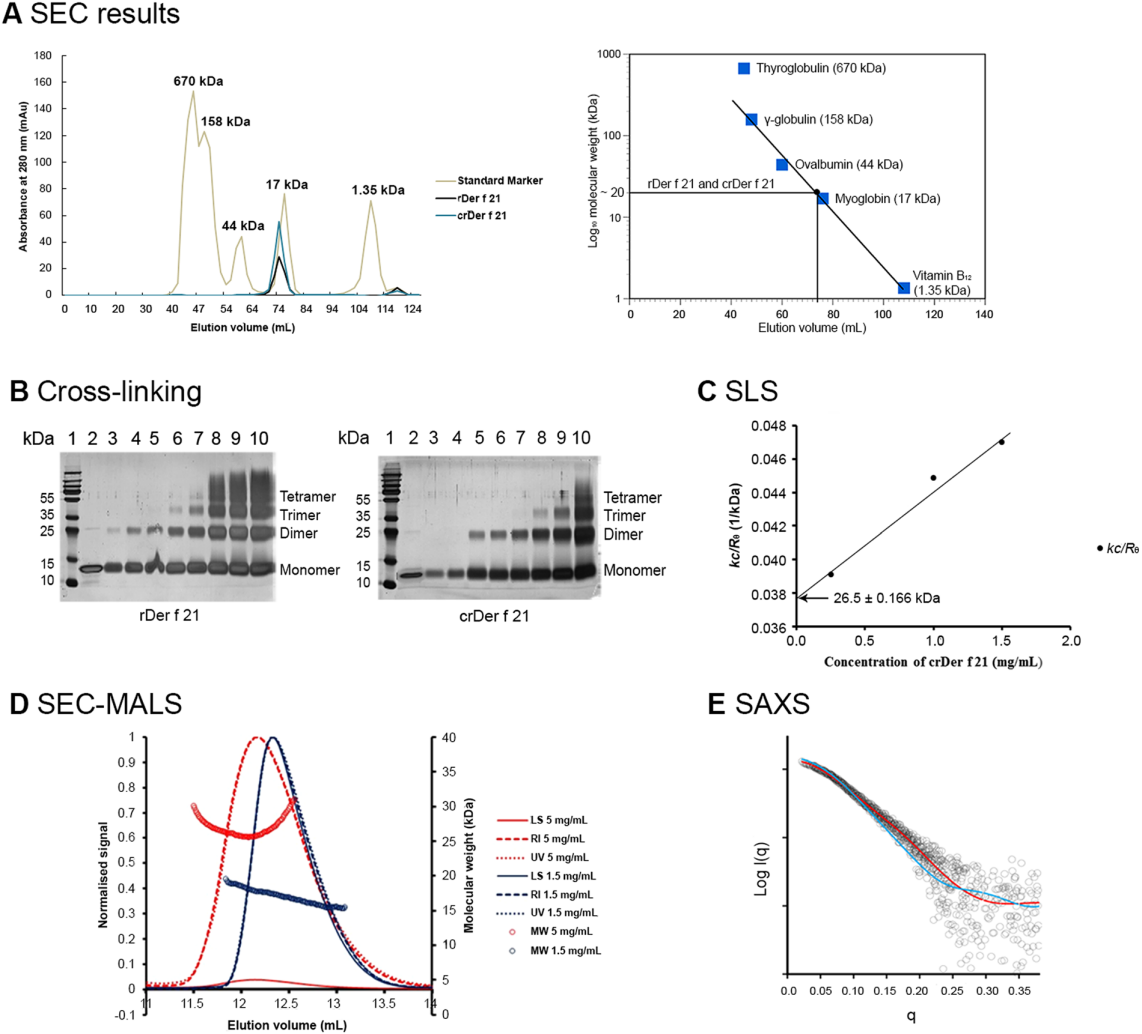
Oligomerisation analysis of rDer f 21. (**A**) Size exclusion chromatography using a HiLoad 16/600 superdex 75 pg shows both rDer f 21 and crDer f 21 eluted at retention volume that corresponded to the molecular weight of ~20 kDa. (**B**) Chemical crosslinking of the rDer f 21 protein and crDer f 21 (6x His-tag was cleaved) with glutaraldehyde (0.002%) for 16 h at 298 K. The crosslinked products were run on 12% SDS-PAGE gel and visualized using silver staining. For both gels: Lane 1: The PageRuler Plus Prestained Protein Ladder, 10–250 kDa (#26619) (Thermo scientific, US); Lane 2: rDer f 21 protein (0.0167 mg/mL) before crosslinking as a negative control; Lanes 3 to 10: Ascending concentrations of cross-linked proteins - 0.0030, 0.0083, 0.0167, 0.0250, 0.0330, 0.0670, 0.1000, and 0.1330 mg/mL. (**C**) Static light scattering data of crDer f 21. The second-virial-coefficient value, *A*_2_, of the crDer f 21 protein is 0.00327 ± 4.27 × 10^−4^, the molecular weight calculated using the equation *k*c/*R*_Ѳ_ = (1/*M* + 2*A*_2_*c*) is 26.5 kDa. (**D**) Size exclusion chromatography with multiangle light scattering analysis of the oligomeric state of rDer f 21. LS: Light Scattering, RI: Refractive Index, UV: Absorbance at 280 nm, MW: Molecular Weight. (**E**) Small angle X-ray scattering analysis of crDer f 21. Red: Monomer, Blue: Dimer.

To investigate the physiological oligomeric state of rDer f 21, and to establish whether the 6x-His-tag fusion promoted dimerization in our crystal structure, the rDer f 21 (15.2 kDa) and crDer f 21 (13.5 kDa) protein samples were prepared and analyzed by SEC, chemical crosslinking, SLS, SEC-MALS, and SAXS studies. The SEC profile shows that both the rDer f 21 and the crDer f 21 proteins were eluted at the same retention volume (~74 mL) with an estimated molecular weight of ~20 kDa. As SEC analysis is also shape dependent, we cannot conclude with high certainty whether rDer f 21 and crDer f 21 proteins form monomers or dimers in solution (Fig. 4A). Crosslinking experiments using a relatively low concentration [0.002% (v/v)] of glutaraldehyde as a crosslinking agent showed that both the rDer f 21 and crDer f 21 proteins could be successfully crosslinked. Both proteins are mainly monomeric at low protein concentration (<0.01 mg/mL), while dimers, trimers and higher oligomers are formed when the protein concentration increases (Fig. 4B). SLS analysis using protein concentrations greater than 0.25 mg/mL have shown that both rDer f 21^21^ and crDer f 21 have molecular weight of 29.5 kDa and 26.5 kDa, respectively, which correspond to the size of the dimer (Fig. 4C). The results obtained from SEC, crosslinking and SLS show that rDer f 21 behaves similarly to crDer f 21 in solution, suggesting that the dimer formation observed in the crystal structure is not an artefact of the N-terminal fusion tag. Interestingly, the SEC-MALS analysis of rDer f 21 at two different concentrations (1.5 mg/mL and 5 mg/mL) shows different molecular weights of ~17.2 kDa and ~32 kDa, respectively (Fig. 4D and Table 2). The MALS data outline that, whilst at the lower protein concentration the molecular weight corresponds to a monomer, at the high concentration rDer f 21 appears to predominantly form a dimer. Both crosslinking and SEC-MALS data indicate that rDer f 21 forms a dimer in a concentration-dependent manner, and it is likely that the obtained crystal structure is a dimer due to the high protein concentration. Nonetheless, the data cannot definitively determine if the biologically functional form of Der f 21 is a monomer, a dimer, or both. Therefore, small angle X-ray scattering measurements were then performed to further determine the oligomerization state of crDer f 21 in solution. Indirect Fourier transformation of the SAXS data determines a protein with a d_max_ of ~57 Å and an R_g_ of 19.5 Å. Because of the small size of the protein, the difference in R_g_ between monomoric (19.7 Å) and dimeric (20.1 Å) state of crDer f 21 is not reliable enough to distinguish between the two states. However, comparison of the calculated SAXS profiles of the monomeric and dimeric forms of crDer f 21 with the solution state SAXS data clearly shows the solution state to be monomeric. (Fig. 4E). Likewise, the particle shape reconstruction from the SAXS data produces a model consistent with the monomeric form of rDer f 21. Overall, the results suggest that Der f 21 may exist as both a monomer and a dimer. Further experiments are needed to confirm its physiological conformation. While various methods have shown that Der f 21 could exist either as a monomer or as a dimer, we have proceeded with the surface-exposed residue mapping and included the residues that reside at the dimer interface region.

**Table 2:** SEC-MALS analysis of rDer f 21.

rDer f 21 concentration (mg/mL)	1.5	5.0
Mn (kDa)	17.2 (±0.3%)	29.1 (±3.5%)
Mw (kDa)	17.2 (±0.3%)	32.0 (±4.8%)
Polydispersity (Mw/Mn)	1.004 (±0.5%)	1.100(±6.0%)

Mn (kDa) is the number-average molecular weight and Mw (kDa) is the weight-average molecular weight.

### Site directed mutagenesis and IgE epitope mapping

**Figure 5:**
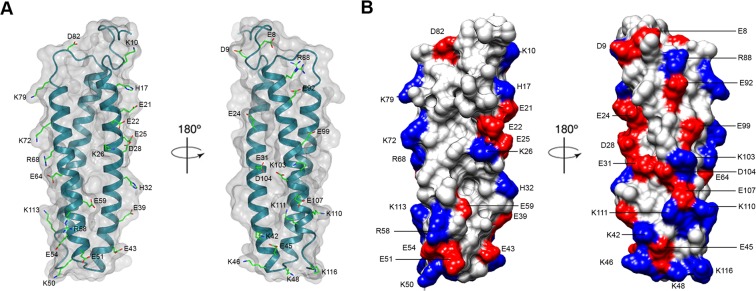
The 38 solvent-accessible, polar and charged amino acid residues selected for the mutagenesis. (**A**) All the selected residues were mapped and shown as cylinder models on the ribbon diagram of rDer f 21. (**B**) Molecular electrostatic surface charge diagram of rDer f 21 with selected residues highlighted. Positively charged and negatively charged residues are colored in blue and red, respectively. Figures **A** and **B** were generated using the program CCP4mg^55^ and the program Chimera^53^, respectively.

The IgE-binding epitopes of protein allergens are mainly composed of the surface-exposed polar amino acid residues^15,16,22^. Previous IgE epitope mapping of dust mite allergens from groups 5, 13, and 21 found that the major IgE epitopes consist of polar and charged residues^15,16,22^. Hence, we identified all solvent-accessible, polar and charged residues (38 in total) on the surface of rDer f 21 including those that reside on the dimer interface, and mutated these residues to alanine to produce a set of 38 single-residue mutants (Fig. 5; Supplementary Fig. S3).

**Figure 6:**
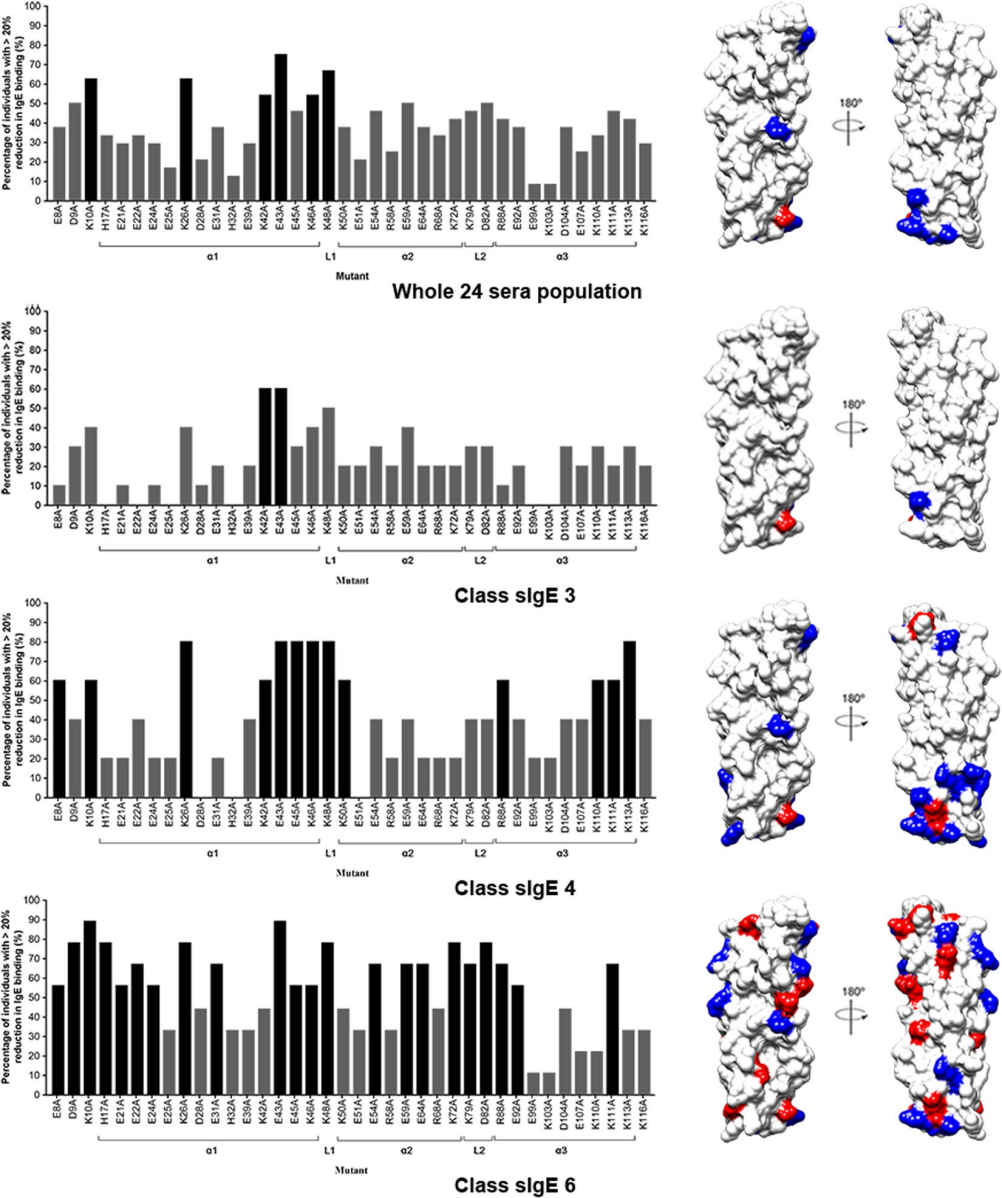
The site directed mutagenesis for the determination of the IgE binding epitopes of rDer f 21. Mutations that cause 20% reduction of the IgE binding in the individual sera are considered significant. Percentage of individuals showing >20% reduction of IgE binding compared to wild-type rDer f 21 was plotted for each mutant. Mutations that cause a significant reduction in the IgE binding in over 50% of individuals (the major epitope residue) are highlighted in black and their location on the rDer f 21 structure is mapped (right panel). Graph (left panel) and surface diagram (right panel) showing the major epitope residues for whole population (24 sera) regardless of their rDerf21-sIgE titres, and classified rDerf21-sIgE sera (class sIgE 3, sIgE 4, and sIgE 6. Blue: positively charged residues; Red: negatively charged residues. The electrostatic surface charge diagram was generated using program Chimera^53^.

To analyse the effect of each mutation on sIgE binding in comparison to the wild type rDer f 21 (set as 100%), the IgE binding capability of each mutant was tested for each of the sera. A mutation was considered as significant if it resulted in more than 20% reduction in IgE binding as compared to the wild type rDer f 21^15,16^. The prevalence of these 38 mutations among 24 atopic individuals is shown in Fig. 6. Of all the 38 residues mutated, only six caused a significant IgE-binding reduction in more than half of the 24 sera. These residues are K10, K26, K42, E43, K46, and K48. Mutation of E43 caused the most drastic reduction in IgE-binding, in which 18 out of 24 sera (75%) showed more than 20% reduction in IgE binding, followed by K48 (16 sera; 67%), K10 and K26 (both 15 sera; 63%), K42 and K46 (both 13 sera; 54%). The other 32 mutants showed IgE-binding reductions for less than 50% of the tested sera (range 8 to 46%). These six residues, K10 (located at N-terminal loop), K26, E42, E43, and K46 (at α1), and K48 (at L1) were therefore identified as the main IgE-binding epitope residues for the rDer f 21 allergen for this tested population of 24 sera.

While most of the sera showed IgE-binding reduction upon mutation of the six main epitope residues, the impact of these mutations varied. As shown in Supplementary Fig. S4, mutation at both residues E43 and K48 caused the most IgE-binding reduction for sera number 7, showing that both E43 and K48 are the main IgE epitope residues for this serum. For sera 11, 14 and 24, IgE binding mainly involved residue K46, with roughly 70% reduction of IgE binding for the single mutant K46A. Residue K26 is the main epitope residue for sera number 4 and 5. The variability of IgE-binding reduction of sera to rDer f 21 mutants suggests heterogeneity in sIgE recognition of the epitopes of the allergen.

### rDerf21-sIgE levels of atopic individuals correlate to sIgE diversity

The 24 rDer f 21–sIgE sera of different IgE titers were subsequently classified using the ImmunoCAP system into three out of a total of six classes, composed of class sIgE 3 (high sIgE level; n = 10), class sIgE 4 (very high sIgE level; n = 5), and class sIgE 6 (very high sIgE level; n = 9). The main IgE-binding epitopes in rDer f 21 were further identified for each sIgE class.

As shown in the Fig. 6, the number of mutations contributing to a significant IgE-binding reduction in more than half of all the 24 sera increases from class sIgE 3 (n = 2) to class sIgE 4 (n = 13), and then to class sIgE 6 (n = 22) (highlighted in black). These main IgE-binding epitope residues were mapped and found to be randomly distributed on the molecular surface of rDer f 21 for sera across the classes sIgE 3 to 6. This result suggests that the higher the rDer f 21-sIgE titers in the sera, the higher the number of main IgE-binding epitopes identified for those respective classes. Hence, the result shows a clear positive correlation between the rDer f 21-sIgE titers and the number of main IgE-binding epitopes for rDer f 21.

## Discussion

The crystal structure of Der f 21 from *D*. *farinae* represents the first crystal structure of a group 21 allergen. Overall, the bundle of three anti-parallel α-helices of rDer f 21 is similar to all available structures of the group 21 and 5 allergens. However, the dimer conformation of rDer f 21, formed via the anti-parallel intermolecular interaction involving helices α1 and α2, is substantially different from the dimer that assembles a hexameric conformation reported for the group 5 allergen, Der p 5. All three α-helices of Der p 5 interact with the helices of the other Der p 5 molecules^12^. Furthermore, the crystal structure of rDer f 21 binds a PEG 400 molecule in the cavity formed at the dimer interface. The binding of the PEG molecule induces a conformational change in helix α1 (residues 20–30) of molecule B of the dimer. Although the rDer f 21 protein structure crystallized with PEG 2K MME as a precipitant neither shows the helical distortion nor binds a molecule of PEG 2K MME, some unidentified weak electron density was also observed in the cavity. The cavity is mostly formed by hydrophobic residues and the binding of PEG 400 is mediated only by three hydrogen bonds through the residue Y66 and two water molecules. A precedent for the hydrophobic cavity in rDer f 21 is shown by the previously reported crystal structure of the Der p 5 dimer. This structure forms a large hydrophobic cavity with a molecule of methylpentanediol bound to it^12^. Interestingly, the hydrophobic cavities of both the Der f 21 and Der p 5 dimers are located in similar positions despite overall differences in the dimer conformation (Supplementary Fig. S5). The existence of a hydrophobic cavity which could accommodate a ligand and also co-localization of all group 5 and 21 allergens in the midgut and hindgut region of the HDM digestive system suggests that group 5 and 21 allergens may perform a similar function^7,8^. The observation of the hydrophobic cavity in rDer f 21 prompted us to hypothesize that the cavity has lipid or lipid-like binding properties similar to Der p 5^12,23^. Furthermore, knowing that only PEG 400 but not PEG 2K MME can fit within the cavity, we further deduce that a molecule of a size similar to PEG400 may exist as a physiologically active molecule for Der f 21. Several families of allergen proteins have been reported to bind lipidic ligands via electrostatic or hydrophobic interactions^24–27^. The binding of immunostimulatory and immunomodulatory lipids has been shown to play a role in protein allergenicity^28^. Although the physiological significance of the dimeric conformation of Der f 21 is yet to be confirmed, we cannot rule out the possibility that under certain circumstances in the cell or in the HDM digestive system, the Der f 21 protein may form a functional dimer, similar to Der p 5 which forms an oligomer at low pH^29^. The binding of a ligand, which could trigger conformational changes in Der f 21 may be important for its biological function. Nonetheless, additional experiments need to be carried out to confirm the physiological function of the observed dimer cavity.

To further investigate whether rDer f 21 is a dimeric protein in solution, the allergen was purified and analysed using multiple approaches. Chemical crosslinking and SEC-MALS showed that formation of the Der f 21 dimer is likely to depend on the protein concentration. SAXS analysis further revealed that the His-tag cleaved crDer f 21 protein exists as a monomer in solution (Fig. 4E). In summary, both the monomeric and dimeric forms of rDer f 21 were observed using a wide range of analytical methods. This phenomenon is consistent for related allergens. In the previous study of group 5 allergens, Der p 5 was crystallized as a hexamer, but was confirmed to form a monomer in solution, with the oligomeric form appearing at high protein concentrations and at low pH^29^. Der p 21 shares ~70% sequence identity to Der f 21 and was shown to be a dimer^8^. Therefore, our results are in accordance with the previous observations that both the monomeric and dimeric conformations can be adopted by the α-helical bundle proteins of the group 5 and 21 HDM allergens.

Allergy symptoms are triggered when IgE antibodies bound on mast cells by the Fc\RI/FcℇRII receptors are crosslinked via epitopes on the allergen surface^30^. Surface-exposed polar or charged amino acid residues are the most abundant IgE epitopes of proteins as shown in previous allergen epitope studies^15,16,22^. With that in mind, we sought to identify the common major epitope residues (see previous section). Our single-site mutagenesis and IgE epitope mapping results identified six main epitope residues (K10, K26, K42, E43, K46, and K48) that are located at the N-terminus, the α1 helix and the L1 loop region of rDer f 21, mutation of which was found to cause significant IgE-binding reduction for more than half of the studied population of 24 atopic sera (Fig. 6). Epitope residue E43 is the most important residue among those sera from sIgE classes 3 to 6 (Fig. 6). This could make it a promising target in the future design of hypoallergens for treatment of the rDer f 21 allergic individuals.

Further analyses designed to identify the main IgE-binding epitopes in rDer f 21 according to sera-sIgE titres found a direct correlation between the number of major epitopes of the rDer f 21 allergen and the sera sIgE levels. We also observed a random distribution of significant epitope residues on the molecular surface of rDer f 21, suggesting a heterogenous population of sIgE in the sera of atopic individuals in epitope recognition (Fig. 6). This is in accordance with a previous study that identified the correlation between IgE titres and complexity of IgE repertoires against Der p 2 allergen^19^. In addition, previous studies on patients with milk and peanut food allergies showed a heterogeneous pattern of the IgE antibody recognition^31,32^. The correlation between epitope diversity and the severity of allergic reaction among patients with milk and egg allergies has also reported previously^31–37^. Atopic individuals that are positive to rDer f 21 with higher levels of rDerf21-sIgE also have higher diversity of sIgEs (Fig. 6). Interestingly, we also see that the number of major epitope residues increased in the studied population from a low to a high sIgE level. This suggests that the profile of major epitopes of an allergen could vary between atopic individuals, and shows that the major epitope residues of an allergen could be further classified according to the sIgE level of atopic population. Although a common major epitope can be identified for an allergen through pooled sera, our results indicate that a more precise major epitope identification of an allergen for an atopic individual can be achieved by knowing their sIgE levels. This finding provides important information for (1) the development of more precise diagnostic methods that could identify specific sIgE epitopes of an allergen for personal allergy diagnosis and (2) the improvement of personal allergen-specific immunonotherapy design with the aim to increase the efficiency of allergy immunotherapy (AIT).

The structural and epitope studies presented here provide a greater understanding of the features of the groups 21 and 5 allergens, including the characterisation of the potential ligand binding cavity that undergoes conformational changes upon ligand binding. The extensive structure-guided mutagenesis and the immuno-dot blot analysis revealed that atopic individuals have heterogeneity in sIgE recognition of the epitope of the allergen, and that the sIgE level of an atopic individual is positively correlated to the number of major epitope residues that sIgE can recognise. This insight provides supporting evidence for the further investigation of whether other aeroallergens or major allergens show a similar correlation between the sIgE level and the number of major epitopes. This has a great importance for the future development of improved personalized allergen diagnosis and AIT treatment. We propose that, applying a refined molecular diagnostic assay using a full set of recombinant allergen, with all surface-exposed residues mutated in turn, to the sera of atopic individuals with a defined sIgE level from class 1 to 6 (etc: ImmunoCAP system) could help to identify more person-specific major epitopes, which could provide another dimension in allergy diagnosis.

## Methods

### Isolation of a novel allergen Der f 21 from *Dermatophagoides farinae*

Der f 21 (GenBank Accession Number AY800349) was isolated from *D*. *farinae* using an expressed sequence tag (EST) developed in- house^38^. Der f 21 shares ~36% amino acid sequence identity with Der f 5 (GenBank Accession Number BAE 45865)^38^. Amino acid residues 1-17 were predicted as a signal peptide using the *SignalP* 4.1 server^39^.

### Cloning, expression and purification of recombinant Der f 21 (rDer f 21 and crDer f 21)

Der f 21 (GenBank Accession Number AY800349; AHC94506) was cloned in pET-M (a modified pET-32 (a) vector)^20^. The His-tagged rDer f 21 protein was then over-expressed using an *E*.*coli* expression system, and purified using Ni-NTA affinity and size-exclusion chromatography (SEC) as described previously^20^. The N-terminal 6x-His-tag of purified rDer f 21 was cleaved using thrombin (10 unit/mg of protein). Cleaved rDer f 21 (crDer f 21) protein was eluted from the Ni-NTA affinity column in the flow-through fraction, which was subsequently loaded on SEC column as an extra purification step. Both the rDer f 21 protein with uncleaved His-tag and His-tag cleaved crDer f 21 were further used for a range of experiments.

### Crystallization and X-ray diffraction data collection

Two different crystal forms suitable for data collection were obtained from purified His-tagged rDer f 21 concentrated to 10 mg/mL. The first type of His-tagged rDer f 21 protein crystals grew from 0.19 M Tris (pH 8.0) and 32% PEG 400. These crystals were used for in-house data collection using the Rigaku X-ray generator with a copper anode, MicroMax-007 HF, coupled with the VariMax HF optic and R-AXIS IV++ IP area detector^20^. The data from the second type of the His-tagged rDer f 21 protein crystals grown from the reservoir solution containing 0.1 M Tris (pH 8.0) and 30% PEG 2000 MME were collected on the Pilatus 6 M detector at the beamline I02 at the Diamond Light Source, UK.

### Structure determination, model building and refinement

The diffraction data from the His-tagged rDer f 21 crystals grown from the solution containing PEG 400 (PEG400) were processed as described previously^20^. The data from crystals grown using PEG 2000 MME (PEG2KMME) as a precipitant were processed using XDS^40^ in space group P2_1_. Data from both crystals were indexed with iMOSFLM^41^, and scaled and merged with the program AIMLESS^42^. The rDer f 21^PEG400^ structure was solved by molecular replacement using the program PHENIX^43^ with residues 66–127 of the dust mite allergen Der p 5 (PDB code 3MQ1) as a search model^12^. The rDer f 21^PEG2KMME^ structure was determined by molecular replacement with PHASER^44^ using rDer f 21^PEG400^ as the search model. The structural models were built with the program ARP/wARP^45^. Further refinement and model building were performed using Refmac5^46^ and COOT^47^. Finally, both rDerf 21 structures were validated using the Ramachandran plot^48^. Data collection, processing and refinement statistics are summarized in Table 1. The structures were deposited in PDB with accession codes of 5YNX (rDer f 21^PEG400^) and 5YNY (rDer f 21^PEG2KMME^).

### Oligomerization studies of rDer f 21

#### Chemical cross-linking

The purified rDer f 21 and crDer f 21 protein solutions (0.0030, 0.0083, 0.0167, 0.0250, 0.0330, 0.0670, 0.1000, and 0.1330 mg/mL) in PBS buffer (30 *µ*L) containing 1.8 mM KH_2_PO_4_ (pH 7.4), 0.01 M Na_2_HPO_4_, 0.14 M NaCl and 2.7 mM KCl were incubated with 0.002% (v/v) glutaraldehyde (Merck) at 298 K for 16 hours. The cross-linking reactions were quenched by the addition of SDS-PAGE sample buffer and were electrophoresed on a 12% SDS-PAGE gel, and the protein bands were visualized using silver staining.

#### Static light scattering

As described previously, rDer f 21 (0.50, 1.08, and 1.61 mg/mL) was prepared for the static light scattering (SLS) experiment^20^. The same experiment was also performed with the crDer f 21 protein samples in three different concentrations (0.25, 1.00, and 1.50 mg/mL).

#### Size exclusion chromatography with multiangle light scattering

Size exclusion chromatography with multiangle light scattering (SEC-MALS) experiments were performed for rDer f 21 using a Superdex S75 increase column (GE Healthcare) on an AktaPure 25 System (GE Healthcare) for rDer f 21. Two 100 *μ*L protein samples (1.5 mg/mL and 5 mg/mL, respectively) were loaded onto the gel filtration column and eluted with one column volume (24 mL) of the elution buffer containing 20 mM Tris (pH 7.9) and 50 mM NaCl, at a flow rate of 0.7 mL/min. The eluted protein was monitored using a DAWN HELEOS-II 18-angle light scattering detector (Wyatt Technologies) equipped with a WyattQELS dynamic light scattering module, a U9-M UV/Vis detector (GE Healthcare), and an Optilab T-rEX refractive index monitor (Wyatt Technologies). Data were analysed by using Astra (Wyatt Technologies) using a refractive increment value of 0.185 mL/g.

#### Small Angle X-ray Scattering

Small angle X-ray scattering (SAXS) experiments were performed at the beamline B21 (Diamond Light Source, Didcot UK). Due to the small size of rDer f 21, size exclusion chromatography-coupled SAXS, (SEC-SAXS), was performed using a capture mechanism on an Agilent 1260 HPLC instrument with a UV-absorbance detector at 280 nm. Briefly, an HPLC valve was placed immediately prior to the SAXS flow cell with a low pressure check-valve attached on the exit side of the cell. A small scale, analytical, SEC run was performed by injecting ~1 mg/mL of protein (45 *μ*L) onto a 4.8 mL KW-402.5 column (Shodex) at 0.25 mL/min. A single elution peak was observed and the corresponding elution time of the protein of interest was noted. A larger scale SEC separation was also performed by injecting ~10 mg/mL of protein. At approximately one second after the peak elution time, the valve was switched to divert the SEC flow thereby capturing the protein in the SAXS cell. The specialized SAXS flow cell has a 1.6 mm path length and was held at 293 K. SAXS measurements were made using a sample to detector distance of 4.09 m and X-ray wavelength of 1 Å. For the protein, a single 3-minute exposure was performed as a collection of 18 × 10 second exposures (frames). After data collection, the valve was switched back to the cell and the contents of the cell were sent to waste. Following ~10 minutes of flow, the valve was switched again and a corresponding measurement was made of the buffer for background subtraction.

Raw images were normalized and integrated into 1-dimensional scattering curves using in-house beamline software (GDA). Background subtraction and data reduction were performed with ScÅtter (http://www.bioisis.net/tutorial). Model fitting of the X-ray crystal structure was performed with CRYSOL and *ab initio* modelling was performed with GNOM and DAMMIF/N^49^.

#### Generation of rDer f 21 mutants

The program AREAIMOL of the CCP4 suite^50^ was used to calculate the residue solvent accessibility (ASA) of both the monomer (molecule A of the dimer) and the dimer of the rDer f 21 proteins. The obtained ASAs were normalized to the corresponding Gly-X-Gly (GXG) tripeptide for more accurate calculation^51^. The ratio of ASA to calculated GXG value of polar charged amino acid residues was obtained for molecule A of both the monomer and the dimer. A total of 38 residues were selected to be mutated to alanine. The 38 new constructs were synthesized, cloned in pET 28 b (+) (GenScript, Piscataway, New Jersey, USA) and transformed into *Escherichia coli* strain BL21 (DE3) cells to produce N-terminal 6x-His-tag fusion protein. Transformed bacterial cells were grown overnight at 310 K in Luria-Bertani (LB) medium containing kanamycin (50 *μ*g/mL). His-tagged rDer f 21 mutants were then expressed and purified using Ni-NTA affinity chromatography as described previously^21^.

#### Screening of sera of atopic individuals by immuno-dot blot

One microgram of *D*. *farinae* crude extract and rDer f 21 proteins (wild type and 38 mutants) were dotted in duplicate onto nitrocellulose membrane together with a serial dilution of IgE standard (1000 IU/mL serially diluted two fold to 1.95 IU/mL; National Institute for Biological Standards, United Kingdom) to provide a standard curve. One microgram of bovine serum albumin (BSA) and 1 *µ*L of protein buffer were used as a negative protein control and a negative control, respectively. The membrane was air-dried, blocked with PBS-T 0.1% (1 X phosphate buffered saline with 0.1% Tween 20) and then incubated overnight with sera (1:4 in PBS) at 277 K. After the washing step, the membrane was incubated with anti-human IgE antibodies conjugated with alkaline phosphatase (1:1000 in PBS; Sigma Aldrich St. Louis Missouri, USA). The alkaline phosphatase activity was detected by addition of nitroblue tetrazolium/5-bromo-4-chloro-3-indolyl-phosphate chromogenic substrate for 10 minutes (Thermo Fisher Scientific, Wathma, MA). Subsequently, spot intensities (range, 0-255) on the membrane were measured using the imaging software (Syngene, United Kingdom). Spot intensities were then averaged and normalized by subtracting the local background. Intensities above the mean negative sera responses were considered positive. The assay performed in this study have been previously used and validated in other publication^52^.

A total of 24 atopic sera that had positive specific IgE binding to both crude *D*. *farinae* extract and rDer f 21 were obtained by immuno-dot blot assay and used to further screen for rDer f 21 epitope residues. All human studies invoved in this report had been reviewed and approved by the Universiti Tunku Abdul Rahman Scientific and Ethical Review Committee (SERC) (U/SERC/03/2015 and U/SERC/03/2016). Signed informed consents were obtained from all volunteers to obtain blood samples. All experiments were performed in accordance with the relevant guidelines and regulations of the institutions and committees indicated above.

## Supplementary information

Supplementary Information
