# Supplementary material for: Crystal structure and epitope analysis of house dust mite allergen Der f 21
Source: Sci Rep. 2019 Mar 20;9:4933. doi: 10.1038/s41598-019-40879-x (PMC6426935; doi:10.1038/s41598-019-40879-x)
Supplement: Supplementary file 1 — Supplementary Information [file 41598_2019_40879_MOESM1_ESM.pdf]

Supplementary information

Crystal structure and epitope analysis of house dust mite allergen Der f 21

Sze Lei Pang[a], Kok Lian Ho[b], Jitka Waterman[c], Robert Paul Rambo[c], Aik-Hong Teh[d], Indran Mathavan[e,f], Gemma Harris[f], Konstantinos Beis[e,f], Yee-How Say[g], Matta Sri Anusha[h], Yang Yie Sio[h], Fook Tim Chew[h]* and Chyan Leong Ng[a]*

[a]Institute of Systems Biology, Universiti Kebangsaan Malaysia, 43600 UKM Bangi, Selangor, Malaysia

[b]Department of Pathology, Faculty of Medicine and Health Sciences, Universiti Putra Malaysia, 43400 UPM Serdang, Selangor, Malaysia

[c]Diamond Light Source, Harwell Science & Innovation Campus, Didcot, Oxfordshire, OX11 0DE, UK

[d]Centre for Chemical Biology, Universiti Sains Malaysia, 10 Persiaran Bukit Jambul, 11900 Bayan Lepas, Penang, Malaysia

[e]Department of Life Sciences, Imperial College London, South Kensington, London SW7 2AZ, United Kingdom

[f]Research Complex at Harwell, Rutherford Appleton Laboratory, Oxfordshire, OX11 0FA, United Kingdom

[g]Department of Biomedical Science, Faculty of Science, Universiti Tunku Abdul Rahman (UTAR) Perak Campus, 31900 Kampar, Perak, Malaysia

[h]Department of Biological Sciences, National University of Singapore, 14 Science Drive 4, 117543, Singapore

*corresponding authors email: clng@ukm.edu.my and dbscft@nus.edu.sg

Supplementary Table S1

Interface analysis of rDer f 21 structure

Interaction	Molecule A		Molecule B		Length (Å)
	Residue	Side chain	Residue	Side chain	
Hydrogen bond	R5	NH1	Q55	OE1	3.09
	R12	O	Q55	NE2	3.75
	N13	OD1	Q55	NE2	2.90
	A14	N	E59	OE2	2.78
	F15	N	E59	OE1	2.82
	Q55	OE1	R5	NH1	3.20
	Q55	NE2	R12	O	3.81
	Q55	NE2	N13	OD1	2.95
	E59	OE1	F15	N	2.85
	E59	OE2	A14	N	2.73
	E77	OE2	N65	ND2	2.95
Salt bridge	E22	OE1	K26	NZ	2.80
	E22	OE2	K26	NZ	3.83

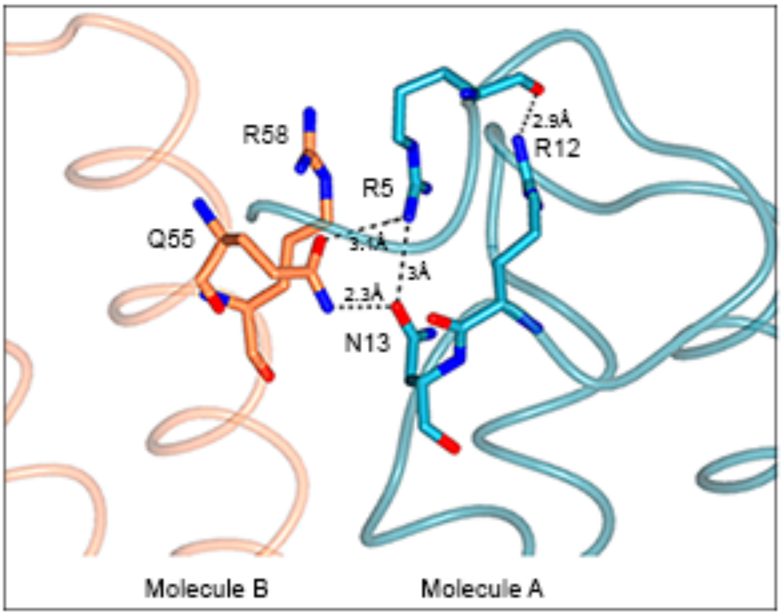

Supplementary Figure S1. The dimer interface of rDer f 21^{PEG400} contributed by residue R5. Hydrogen bonds involving R5 and arginine stacking (R5^{A}, R12^{A} and R58^{B}) are shown. The figure was generated using CCP4mg[1].

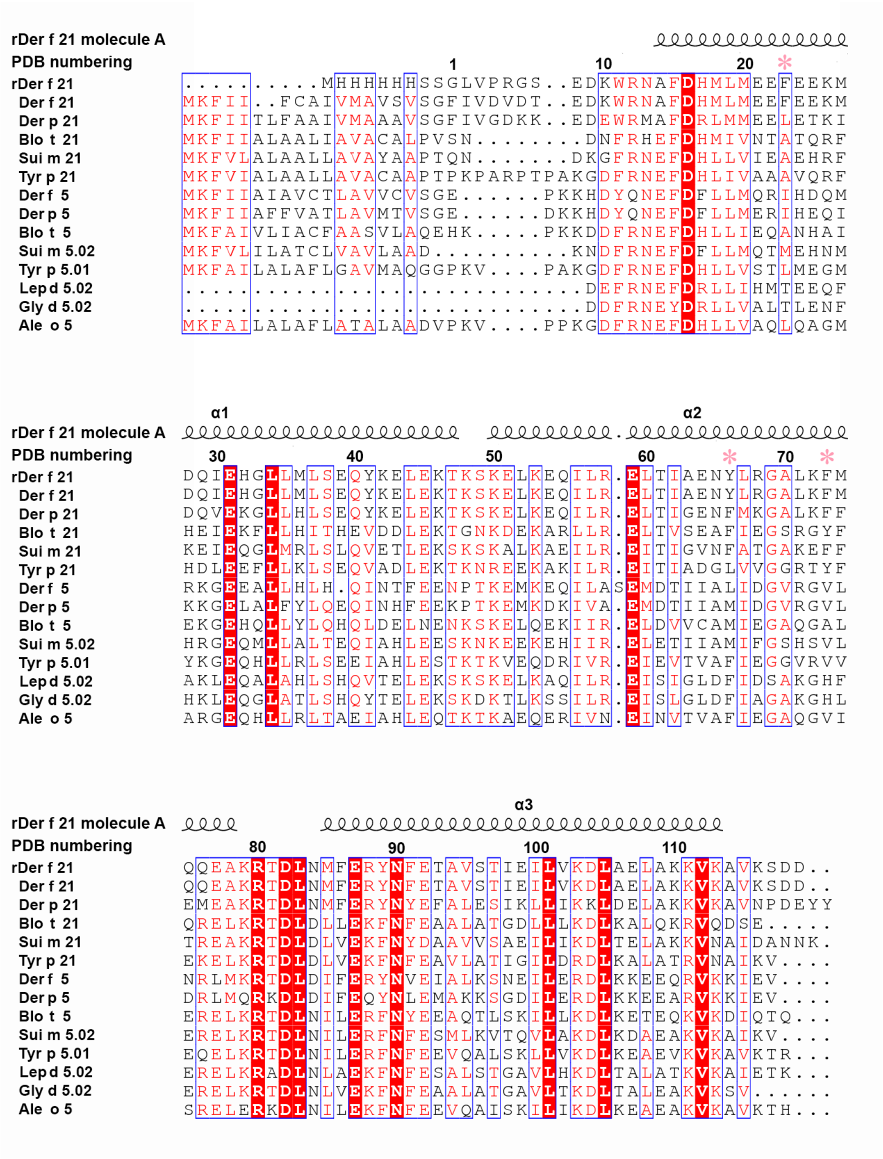

Supplementary Figure S2. Comparison between rDer f 21 protein sequence and homologous allergens. The alignment of the rDer f 21 with complete amino acid sequence of Der f 21, the HDM allergens (Der p 21, Blo t 21, Der f 5, Der p 5 and Blo t 5), and storage mite allergens (Sui m 21, Tyr p 21, Sui m 5.02, Tyr p 5.02, Lep d 5.02, Gly d 5.02 and Ale o 5). The conserved amino acid residues are coloured in white with red background. The secondary structure elements from the rDer f 21 crystal structure (helices α1, α2 and α3) are highlighted above the alignment. The numbering of rDer f 21 according to the pdb file is shown on the top. The aromatic residues (F23, Y66 and F73) involved in the formation of the rDer f 21 binding cavity are indicated with a pink star. The sequence alignment was performed using ClustalW[2] and the figure was prepared using ESPript 3.0[3].

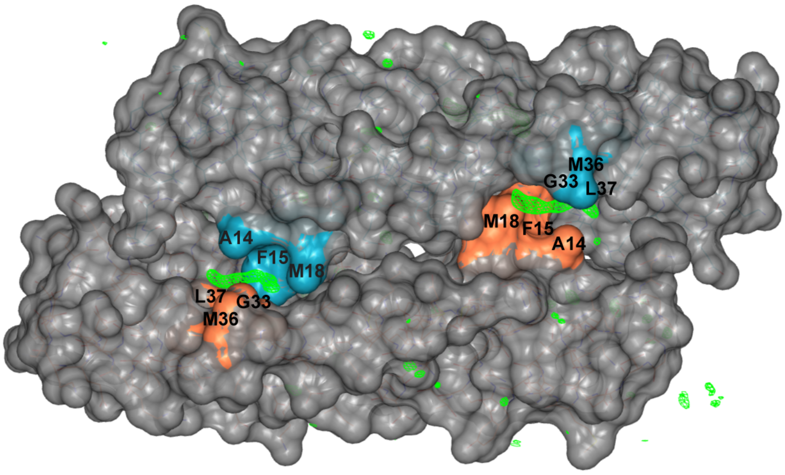

Supplementary Figure S3: Unidentified regions of small molecule electron density were found in two cavities formed by hydrophobic residues of rDer f 21^{PEG400} dimer interface. The surface in cyan and coral represents molecule A and B of rDer f 21^{PEG400}, respectively. The Fo - Fc difference electron density map was contoured at 3.5 σ. The figure was generated using the molecular-graphics program CCP4mg[1].

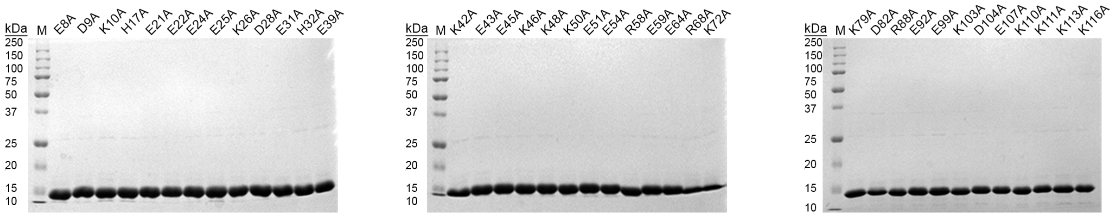

Supplementary Figure S4. Sodium dodecyl sulfate - polyacrylamide gel electrophoresis (SDS-PAGE) of the 38 solvent-accessible, polar and charged amino acid residues selected for the mutagenesis. 12.5% SDS-PAGE gels showing all 38 mutant proteins after purification using affinity chromatography.

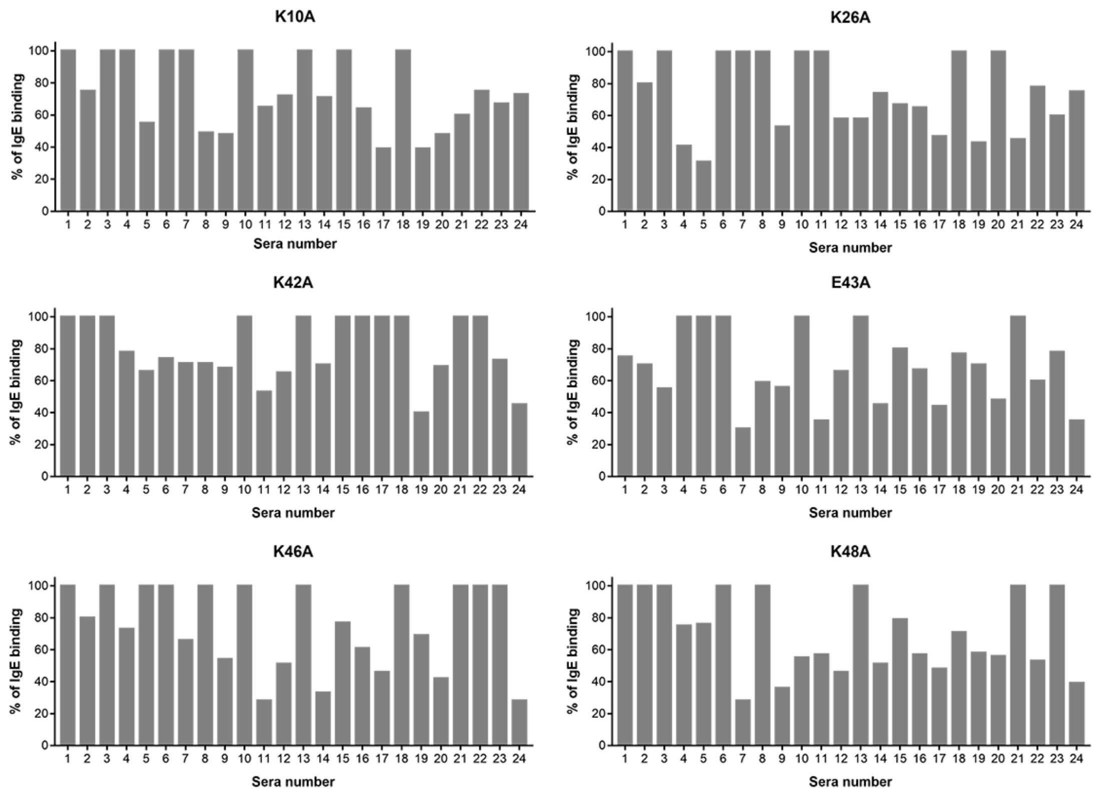

Supplementary Figure S5. IgE binding of 24 sera to six rDerf21-major IgE epitopes was determined using immuno-dot blot assay. Percentage of IgE binding to each mutant have been normalised to percentage of IgE binding to wild type rDer f 21 (set as 100%) for each individual sera. The sera that showed 100% of IgE binding or no IgE-binding reduction to one mutant means that the particular mutant is not an epitope for the sera. Six major IgE epitopes were identified for rDer f 21 (K10, K26, K42, K43, K46, and K48). Mutation of these six major IgE epitopes resulted in variable percentage of IgE-binding reduction among different individuals.

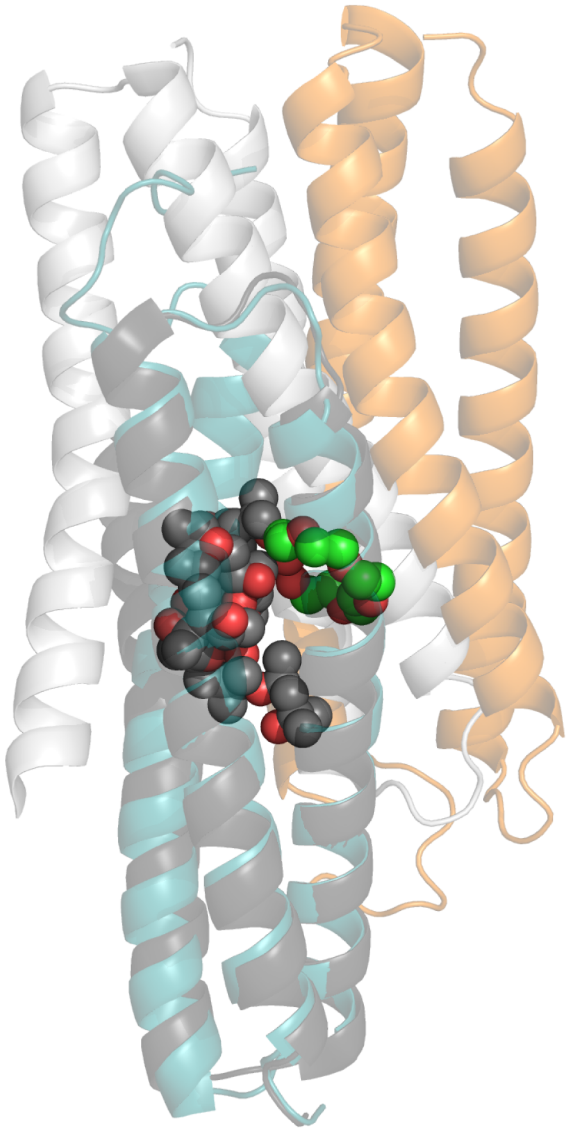

Supplementary Figure S6. Superimposition of the rDer f 21 and Der p 5 structures shows their conserved cavity regions with a PEG 400 molecules (green spheres) and methylpentanediol (black spheres) bound, respectively. Both ligands are bound at a similar coordination despite the overall conformation of each dimer being different. Both rDer f 21 and Der p 5 dimers are presented as a cartoon diagram. Molecules A and B of rDer f 21 are coloured cyan and orange, respectively. Molecules A and B of Der p 5 are coloured grey (molecule A) and white (molecule B). The figure was generated using Pymol[4].

References

1. McNicholas, S., Potterton, E., Wilson, K. & Noble, M. Presenting your structures: the CCP4mg moleculargraphics software. Acta Crystallogr D Biol Crystallogr 67, 386–394 (2011).

2.Thompson, J. D., Gibson, T. & Higgins, D. G. Multiple sequence alignment using ClustalW and ClustalX. Curr Protoc Bioinformatics 1, 2–3 (2002).

3. Robert, X. & Gouet, P. Deciphering key features in protein structures with the new ENDscript server. Nucleic Acids Res 42(W1), W320–W324 (2014).

4.DeLano, W. L. The PyMOL molecular graphics system, http://www.pymol.org (2012).
